# The *N-*glycosylation at positions 652 and 661 of viral spike protein negatively modulates porcine deltacoronavirus entry

**DOI:** 10.3389/fvets.2024.1430113

**Published:** 2024-05-30

**Authors:** Hai-Ming Wang, Yang-Yang Qiao, Yong-Gang Liu, Bing-Yan Cai, Yue-Lin Yang, Hui Lu, Yan-Dong Tang

**Affiliations:** ^1^Jiangsu Agri-animal Husbandry Vocational College, Taizhou, China; ^2^State Key Laboratory for Animal Disease Control and Prevention, Harbin Veterinary Research Institute of Chinese Academy of Agricultural Sciences, Harbin, China; ^3^Heilongjiang Provincial Research Center for Veterinary Biomedicine, Harbin, China

**Keywords:** PDCoV, *N-*glycosylation, entry, pseudotyped virus, spike protein

## Abstract

*N*-glycosylation is a highly conserved glycan modification that plays crucial roles in various physiological processes, including protein folding, trafficking, and signal transduction. Porcine deltacoronavirus (PDCoV) poses a newly emerging threat to the global porcine industry. The spike protein of PDCoV exhibits a high level of *N*-glycosylation; however, its role in viral infection remains poorly understood. In this study, we applied a lentivirus-based entry reporter system to investigate the role of *N*-glycosylation on the viral spike protein during PDCoV entry stage. Our findings demonstrate that *N*-glycosylation at positions 652 and 661 of the viral spike protein significantly reduces the infectivity of PDCoV pseudotyped virus. Overall, our results unveil a novel function of *N*-glycosylation in PDCoV infection, highlighting its potential for facilitating the development of antiviral strategies.

## Introduction

The Porcine deltacoronavirus (PDCoV) is classified as a member of the *Deltacoronavirus* genus within the *Coronaviridae* family, which belongs to the order Nidovirales (1). It was first reported in 2012 (2), and since then it has emerged as a significant threat to the swine industry, inducing gastrointestinal symptoms in piglets, potentially leading to dehydration and fatal outcomes (3–5). Furthermore, recent evidence suggests that PDCoV can cross species barriers and infect humans, highlighting its potential as a public health concern (6).

*N*-glycosylation is a highly conserved glycan modification that plays pivotal roles in diverse biological processes, including protein folding, quality control, glycoprotein interaction, intracellular trafficking, signal transduction, viral attachment, and immune response to infection (7). The biosynthesis of *N*-glycans involves a complex cascade of events occurring within both the Endoplasmic Reticulum (ER) and the Golgi apparatus. Within the ER, *N*-glycan precursors are synthesized and subsequently transferred to the consensus sequence N-X-S/T (where X represents any amino acid except proline) on nascent proteins (8, 9). During viral replication, *N*-glycosylation of viral glycoproteins plays multiple roles, including evading antibody neutralization, interacting with cell surface receptors, and facilitating fusion events (10).

The cryo-electron microscopy structure of the Spike (S) glycoprotein has recently been elucidated, revealing a prominent *N*-glycosylation pattern on PDCoV S protein (11, 12). However, the role of *N*-glycosylation in PDCoV infection remains unexplored. In this study, we investigated the impact of *N*-glycosylation on viral entry by a PDCoV pseudotyped virus assay (13). The pseudotyped virus is characterized by single-cycle infection, which exhibits infectivity without the ability to proliferate. Various approaches have been employed for generating single-cycle infection viruses; however, the lentivirus-based pseudotyped virus is widely applied (14–16). Our aim was to elucidate whether *N*-glycosylation in the viral spike protein is involved in virus entry. Our findings indicate that *N*-glycosylation at positions 652 and 661 plays a negative role in PDCoV entry. Glycan modification at these sites significantly reduced the infectivity of the pseudotyped virus at viral entry stage. Furthermore, our study provides valuable insights into potential targets for antiviral interventions against PDCoV.

## Materials and methods

### Cells, virus, reagent, and plasmids

The PK15, LLC-PK, and HEK293T cell lines were cultured in DMEM (Gibco, United States) supplemented with 10% fetal bovine serum (HyClone, United States). The PDCoV S gene was cloned into the pCAGGS-HA vector as previously described (13). The spike protein with N652Q and N661Q were constructed by site-directed mutagenesis using PCR (17, 18). The specific antibody against the S protein was generously provided by Prof. Shaobo Xiao from Huazhong Agriculture University.

### Western blotting

HEK293T cells were transfected with 2 μg of plasmids encoding wild-type S, N652Q, or N661Q variants, respectively. After 48 h, the cells were lysed and subjected to Western blot analysis using a previously described procedure outlined in our previous publications (19–24).

### Pseudovirus entry assay

The PDCoV pseudotyped virus was generated in HEK293T cells using previously described methods (13, 15, 18). Briefly, HEK293T cells were seeded in 6-well plates and co-transfected with HIV-1-based luciferase reporter plasmids (3 μg), along with packaging plasmids psPAX2 (2 μg) and PDCoV-S (1 μg), to produce pseudotyped viruses (17, 18). Plasmids were transfected by calcium phosphate transfection agent. After an incubation period of 8 h, the cells were washed with PBS and subsequently supplemented with serum-free medium. The supernatant containing the pseudotyped viruses were harvested at 48 h post-transfection, from which a volume of 100 μL was used for infecting LLC-PK and PK15 cells. Following a wash step, viral entry was analyzed by Promega luciferase assay at 24 h post-infection (hpi), as previously described (18, 25–27).

### Structural analysis

The Protein Data Bank (PDB) accession code for the PDCoV spike structure model was 6BFU^1^ (11). The structural figures were generated using UCSF Chimera with the help of Dr. Jianbo Liu (28).

### Statistical analysis

The software GraphPad Prism 8.0 was utilized for generating all graphical representations in this study. Statistical significance was assessed using Student’s *t*-test. A *p*-value less than 0.05 was considered statistically significant.

## Result and discussion

### *N*-glycosylation at positions 652 and 661 decreases the infectivity of pseudotyped PDCoV entry

**Figure 1:**
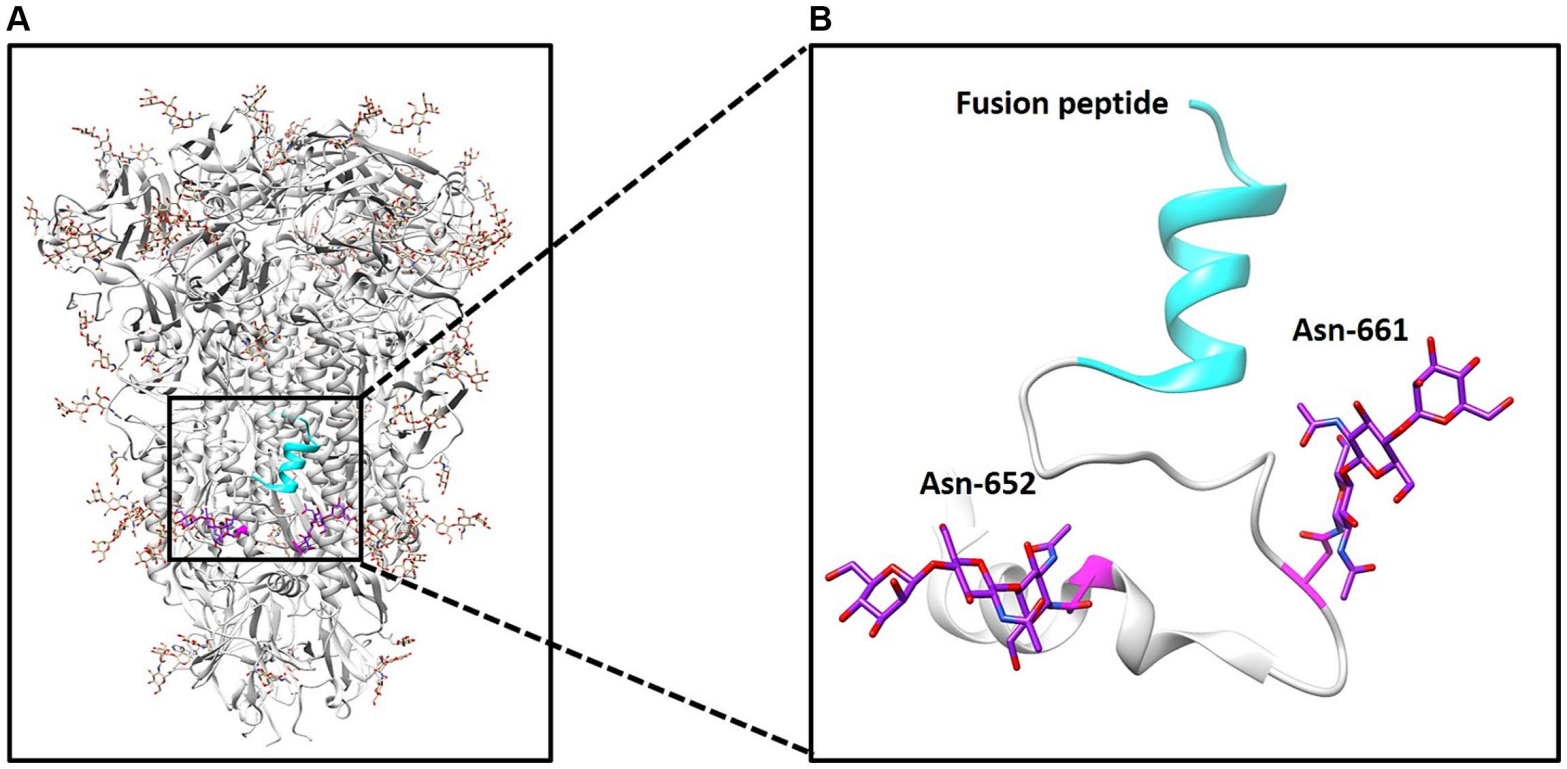
Structure of PDCoV spike protein. **(A)** Structure of spike trimer and *N*-linked glycans at surface are illustrated. **(B)** N-linked glycans at positions 652 and 661 and fusion peptide was illustrated.

The S glycoprotein of the coronavirus facilitates viral entry and acts as a primary determinant of cell tropism and pathogenesis (29). Classified as a class I fusion protein, the S glycoprotein is responsible for receptor binding and mediating fusion between host and viral membranes. Upon receptor binding, the conformation of the S protein undergoes changes, leading to fusion peptide-driven fusion between viral and cellular membranes, initiating membrane fusion between the virus and host. Two studies have employed cryo-electron microscopy to determine the structure of PDCoV’s S protein (11, 12). As illustrated in Figure 1A, the PDCoV S protein forms a compact trimeric assembly. Similar to other coronaviruses, *N*-linked glycans are densely distributed on the surface of PDCoV’s S protein (Figure 1A). The *N*-glycosylation of viral S proteins plays a crucial role in evading neutralizing antibodies, interacting with cell surface receptors, and facilitating fusion events (10). Notably, two glycans at positions 652 and 661 are located near the putative fusion peptide (Figure 1B).

**Figure 2:**
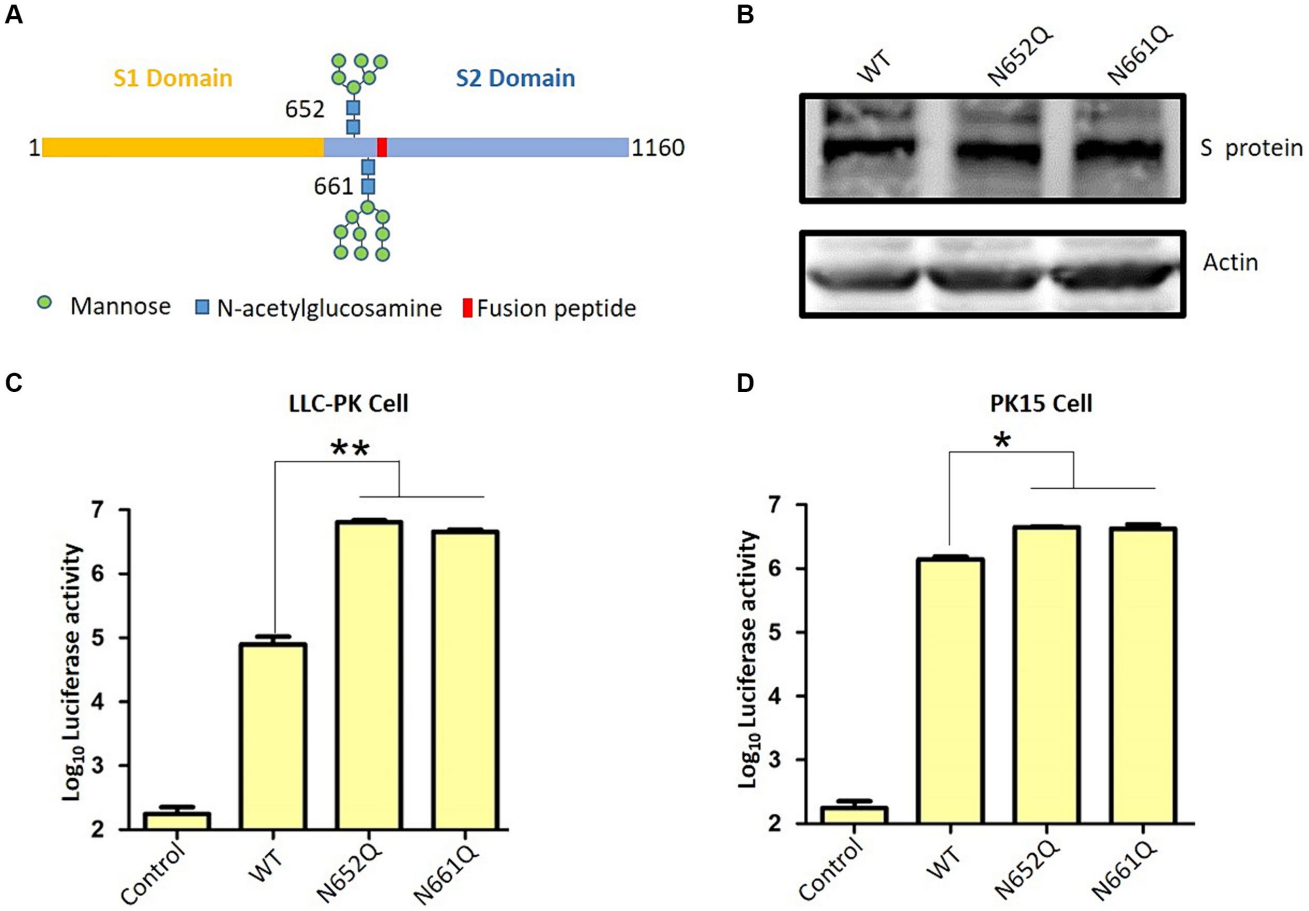
PDCoV spike protein schematic. **(A)** S protein consists of S1 and S2 domain, and 652 and 661 near the fusion peptide is illustrated. **(B)** The expression of wild type S and indicated S mutants. **(C)** Pseudovirus infection in LLC-PK cells and **(D)** PK15 cells. LLC-PK cells or PK15 cells were infected with the same dose of pseudovirus. After 24 h, the cells were lysed and used for luciferase detection.

The S protein ectodomain can be divided into the S1 and S2 domains, and S1 including the receptor binding domain responsible for interacting with the host cell receptor. The S2, known as the fusion peptide-containing region, is critical for initiating membrane fusion between the virus and host cells (Figure 2A) (29). Here, we want to explore the role of *N*-linked glycans at positions 652 and 661 near the putative fusion peptide. Therefore, we employed a pseudotyped virus to assess viral entry as previously described (13, 15, 25). Initially, two S mutants were constructed: N652Q and N661Q, which eliminated *N*-glycan modification without affecting S expression levels (Figure 2B). Subsequently, lentivirus-based pseudotyped viruses containing firefly luciferase reporter were generated by co-transfecting HIV-1-based luciferase reporter plasmids along with packaging plasmids psPAX2 and indicated PDCoV-S plasmids into HEK293T cells. After incubation for 48 h, pseudotyped viruses harboring specific S proteins were collected and used to infect target cells. Successful generation of pseudotyped viruses were validated by measuring luciferase activity in LLC-PK cells, as background luciferase activity was detected in control group (pseudotyped virus without S) (Figure 2C). Interestingly, both N652Q and N661Q mutants of the pseudotyped virus exhibited significantly increased infectivity compared to the wild type. This suggests that *N*-linked glycans at positions 652 and 661 play a negative role in the infectivity of the pseudotyped virus. To further validate these findings, PK15 cells were infected with the indicated pseudotyped viruses, and the N652Q and N661Q mutants also enhanced the infectivity of the pseudotyped virus (Figure 2D). Coronavirus entry into host cells requires the binding of the S protein to its receptor, leading to conformational changes and subsequent viral membrane fusion with the cell. Our study demonstrates that *N*-linked glycans at positions 652 and 661 negatively affect the step of PDCoV entry. However, it remains unclear how these glycans influence virus binding or fusion with host cells. Additionally, there are several other *N*-glycans on the surface of the PDCoV S protein; further investigation is needed to determine if these glycans regulate virus entry. Aminopeptidase N (APN) has been shown to mediate PDCoV entry in non-susceptible cells (30), (31). In our previous research, we have demonstrated that APN facilitates PDCoV entry into host cells through an endocytic pathway, thereby promoting efficient viral replication (32). The influence of these glycans on virus entry may disrupt S protein binding to its receptor; however, this hypothesis needs to be tested in future studies. We propose that the presence of *N*-linked glycans at positions 652 and 661 likely affects the fusion activity of the S protein for two reasons: firstly, these sites are located near the putative fusion peptide; secondly, while the receptor-binding domain (RBD) is located within the S1 domain, both glycans are present in the S2 domain. These glycans may be less accessible for cleavage by other host proteases such as members of transmembrane protease/serine subfamily (TMPRSS), TMPRSS2 or HAT (TMPRSS11d), which cleave at specific sites on the S protein. Therefore, we hypothesize that *N*-linked glycans at positions 652 and 661 regulate viral infectivity possibly by modulating its fusion activity. Proper cleavage of the S protein is crucial for coronavirus replication; for instance, trypsin plays an important role in PDCoV infection. Our recent work indicated that trypsin promotes the replication of PDCoV by enhancing its S protein-mediated cell-to-cell spreading rather than entry (13). The potential impact of glycan on the cleavage of the S protein requires additional investigation. Furthermore, it is important to validate these findings in live virus in future.

In summary, our study revealed that *N*-linked glycans at positions 652 and 661 exert a negative impact on the infectivity of pseudotyped virus. These findings provide valuable insights for optimizing PDCoV titer in vaccine development or identifying potential therapeutic targets.

## Data availability statement

The original contributions presented in the study are included in the article/supplementary material, further inquiries can be directed to the corresponding authors.

## Author contributions

H-MW: Funding acquisition, Investigation, Methodology, Software, Writing – review & editing. Y-YQ: Data curation, Formal analysis, Funding acquisition, Investigation, Methodology, Validation, Visualization, Writing – original draft. Y-GL: Conceptualization, Supervision, Writing – review & editing. B-YC: Data curation, Resources, Validation, Writing – review & editing. Y-LY: Conceptualization, Investigation, Writing – review & editing. HL: Conceptualization, Supervision, Writing – review & editing. Y-DT: Conceptualization, Resources, Supervision, Writing – original draft, Writing – review & editing.

## Footnotes

^1^http://www.emdatabank.org/
